# Predictors of Bacterial Vaginosis among Pregnant Women Attending Antenatal Clinic at Tertiary Care Hospital in Tanzania: A Cross Sectional Study

**DOI:** 10.24248/eahrj.v5i1.652

**Published:** 2021-06-11

**Authors:** Afrin F. Shaffi, Belinda Balandya, Mtebe Majigo, Said Aboud

**Affiliations:** a Department of Obstetrics and Gynaecology, Muhimbili University of Health and Allied Sciences; b Department of Microbiology and Immunology, Muhimbili University of Health and Allied Sciences

## Abstract

**Background:**

Bacterial vaginosis (BV) is one of the most common genital tract infections in pregnancy associated with an increased risk of pregnancy losses, maternal and perinatal morbidity and mortality. Different social behavioural and obstetric factors can contribute to the development of BV. Determining the predictors of BV could be the best way of identifying women at high risk of developing the disease.

**Methods:**

This was a cross-sectional study conducted between December 2017 and February 2018 to determine the prevalence and predictors of BV among pregnant women attending antenatal Clinic (ANC) at Muhimbili National Hospital (MNH), Tanzania. Participants were recruited using systematic random sampling. For each consented participant, a pretested questionnaire was filled, a pelvic examination was done and a sample was collected. BV was diagnosed using Nugent's score. Data was analysed using Statistical Package for Social Scientists (SPSS) version 23.0. Bivariate and multivariate logistic regression analysis was done to determine factors that were independently associated with BV.

**Results:**

178 (26.7%) pregnant women out of 667 were diagnosed positive for BV. In the bivariate analysis (Table 3), age (COR 1.71; 95% CI, 1.16–2.52), level of education (COR 4.08, 95% CI, 2.84–5.84), gravidity (COR, 1.52, 95% CI; 1.04–2.23), parity (COR 1.69, 95% CI; 1.18–2.42), vaginal douching (COR 2.89, 95% CI; 1.96–4.27), HIV status (COR 9.37, 95%CI; 4.12–21.28), history of STI (COR 2.49 95% CI; 1.46–4.25), LTSP (COR 2.76, 95% CI; 1.68–4.54) and age of first coitus (COR 3.19, 95% CI; 2.24–4.56) were significantly associated with BV. After adjusting for confounders in multivariate analysis, the following risk factors remained significantly associated with BV; age of 21–29 years (AOR, 2.22, 95%CI; 1.45–3.49), primary education level (AOR 3.97, 95% CI; 2.63–5.98), vaginal douching (AOR 3.68, 95% CI; 2.35–5.76), HIV status (AOR 6.44, 95% CI; 2.62–15.88), STI infection (AOR 2.34, 95% CI; 1.25–4.37), more than one LTSP (AOR 2.69, 95% CI; 1.53–4.74) and age of less than 18 years of first coitus (AOR 2.16, 95% CI; 1.42–3.30).

**Conclusion:**

The prevalence of BV in pregnant women was found to be high. Age of less than 30 years, primary education level and below, vaginal douching, HIV infection, STI, more than one lifetime sexual partners and early age of sexual debut were found to be significant predictors of BV. The high prevalence of BV in our population should necessitate policy makers to include screening and treatment of BV in the future policy of antenatal care package, as BV is associated with significant maternal and neonatal morbidity and mortality. Women should also be educated on harmful effects of certain behavioural practices such as vaginal douching that predispose to BV. In addition symptoms of BV such as abnormal vaginal discharge during pregnancy are inconsistent, under reported and often overlooked. Therefore, a high-risk approach can be used for screening and treatment of asymptomatic women.

## BACKGROUND

Bacterial vaginosis (BV) is one of the most common genital tract infections during pregnancy, more so in the African population.^1^ It is a syndrome marked by an increased vaginal PH, milky creamy discharge and amine or fishy odour. It is characterised by a shift in the vaginal flora from the dominant lactobacillus species to a mixed vaginal flora. The number of lactobacilli morphotypes is reduced and the number of anaerobic bacterial morphotypes like Gardnerella vaginalis, Prevotella, Mobilincus species, and Mycoplasma hominis is increased.^2^ with an overgrowth of several anaerobic or facultative bacteria and with a reduction or absence of lactobacillus colonisation. The prevalence of BV among pregnant women varies across the globe due to geographical, socio-economical and clinical factors of the population. In high income countries the prevalence of BV among pregnant women ranges from 9.3% to 17%.^3,4^ In Africa, prevalence of as low as 6.4% in Burkina Faso and as high as 38% in Botswana have been reported.^5,6^

High prevalence has been noted in most Sub-Saharan countries; 38%, 32.5%, 20.6% and 19.4% in Botswana, Zimbabwe, Kenya and Ethiopia respectively.^6–9^

Several studies showing prevalence in different sub groups have been carried out in Tanzania. A study done in Mwanza 22 years back showed the prevalence of BV to be 24% among pregnant women attending Antenatal Clinic (ANC).^10^ Another study of a similar nature done 14 years later at BMC showed an escalating prevalence of 28.5%, however this study only considered women in labour, hence the study cannot be applied to all pregnant women.^11^ A study done by Aboud et al showed prevalence of BV to be 60.6 % in HIV positive pregnant women.^12^

The magnitude and determinants of BV have been observed to be varying from one place to another due to the differences in geographical, socio-economical and clinical characteristics of the study populations. Several factors have been associated with the causation of BV, these include; age, race, socioeconomic status, smoking, vaginal douching, sexual activity, multiple sexual partners, history of current or past Sexually Transmitted Disease (STI) and Human Immunodeficiency Virus infection (HIV).^7,9,11–13^ About 50% of the women with BV are asymptomatic. If symptomatic, commonly present with a malodorous discharge and usually there are no clinical signs of infection in the vaginal mucosa.^2^

Several clinical and microscopic scoring systems for the diagnosis of BV have been validated. The most commonly used are the Amsel's criteria and the gold standard laboratory based Nugent Gram staining evaluation.^14,15^ Slides of vaginal smears are Gram stained and the bacterial morphotypes are quantified and scored as follows: Large Gram-positive rods (*Lactobacillus* scored as 0 to 4), small Gram-variable rods (*Gardnerella vaginalis* scored as 0 to 4) and curved Gram-variable rods (*Mobiluncus species* scored as 0 to 2). Bacterial vaginosis is put on a 10-point scale where: 0–3 is regarded as normal (predominantly *Lactobacillus*), 4–6 as intermediate (mixed flora) and 7–10 positive for BV (no *Lactobacillus*.^15^

Bacterial vaginosis is associated with adverse maternal and perinatal outcomes such intrauterine infections, chorioamnionitis, postpartum endometritis, spontaneous abortion, Pre-Term labour (PTL), Premature Rupture of Membranes (PROM), low birth weight babies, neonatal sepsis and death.^16–18^ Intrauterine infections may occur early in pregnancy or even before pregnancy and remain asymptomatic and undetected for months until PTL or PROM occurs. Preterm labour and delivery are among the most challenging obstetric complications encountered.

Although several studies have been conducted in Tanzania showing prevalence of BV in different sub groups, no recent study has been conducted among pregnant women attending ANC to determine the prevalence and predictors of BV.^10–12^ Given the association of BV and poor pregnancy outcome, it called for need of studying and understanding the situation in the local settings.^16–18^ Determining the predictors of BV could be the best way of identifying women at high risk of developing the disease, screening them early and managing them appropriately. This could have a substantial impact in preventing adverse pregnancy and neonatal outcomes associated with BV which in turn decreases the cost factors, morbidity and mortality rates for both mother and neonate. This study was therefore designed to establish the prevalence of BV and its predictors among pregnant women attending antenatal clinic at Muhimbili National Hospital (MNH).

## MATERIALS AND METHODS

### Study Setting

The study was conducted at ANC at MNH, Dar es Salaam. MNH is the largest tertiary care hospital in the country and is the teaching hospital for Muhimbili University of Health and Allied Sciences (MUHAS). Maternity block has 4 neonatal wards, the labour ward, maternal high dependency unit and 4 maternity wards which can accommodate 40 antenatal and postnatal women each. MNH offers specialised obstetrics services for Dar es Salaam city and suburban population (4 million people) (National population and housing census, 2012). Maternity unit receives women directly from home as well as those referred from almost all regional and district hospitals in Dar es Salaam. The Antenatal clinic is conducted in the maternity block where services are provided daily from Monday to Friday, thus a total of 5 antenatal clinics are conducted in a week. Each of the 4 firms in the department has a specific day of the week to run the ANC. There are 7 consultation rooms and one examination room. About 100 women (20 public and 80 private) attend the clinic on daily basis. About 75% women attend in view of their current pregnancy while 25% are post-natal follow-ups. Most of these women come for a follow-up visit more than once per month, majority of whom are high risk cases, while a few are new attendees. On average at each clinic, about 20 pregnant women are attending for their first time and these are referred from other public hospital, from home or those who attend as private. Among the usual services provided at the ANC are health education given by nurses and record weight and blood pressure readings at each visit. This is done before the patient enters the doctor's room. Routine screening for syphilis, blood groups, Rhesus factor, and haemoglobin level determination are also provided. Counselling and screening for HIV, as well as drugs for the prevention of maternal to child transmission of HIV are provided.

### Study Design

This was an analytical cross-sectional study conducted between December 2017 and February 2018

### Study Duration

The study duration was between December 2017 and February 2018.

### Study Population

All pregnant women attending ANC at MNH during the study period

### Inclusion Criteria

All pregnant women attending ANC clinic at MNH during the study period and women who consented to participate in the study

### Exclusion Criteria

Pregnant women with a history of use of antibiotic in the past 2 weeks, history of ante partum haemorrhage, and PROM.

### Sample Size Estimation

The estimated sample size N was calculated using Fleiss formula.^19^

$$n=\frac{{\left[Z_{\alpha }\sqrt{\left(1+1/m)\bar{p}(1-\bar{p}\right)}+Z_{\beta }\sqrt{p_{0}(1-p_{0})/m+p_{1}(1-p_{1})}\right]}^{2}}{{\left(p_{0}-p_{1}\right)}^{2}}$$

$$\bar{p}=\frac{p_{1}+mp_{0}}{m+1}$$

$$n_{c}=\frac{n}{4}{\left(1+\sqrt{1\frac{2(m+1)}{n\;m|p_{0}-p_{1}|}}\right)}^{2}$$

Where α = alpha, β = 1 - power, n_c_ is the continuity corrected sample size and Zp is the standard normal deviate for probability p. n is rounded up to the closest integer. P_0_ (55/99 or 55.6%) is the proportion of BV in women who douched 2 or more times, P1(82/193 or 42.5%)) is the proportion of BV in women who did not douche. Sample size, N was calculated using the study titled “Maternal Stress is Associated with Bacterial Vaginosis in Human Pregnancy” Maternal and Child Health Journal, odds ratio for overall BV was 2.4, 87% power, level of significance 0.05, ratio of sample size was 193/99 or 1.9.^20^ Therefore, N using Open Epi and Fleiss formula was 603, which becomes 663 after taking care of 10% of non-respondents.

### Sampling Technique

Recruitment of participants was conducted exclusively during clinic days. Systematic random sampling technique was used to sample the participants meeting the inclusion criteria. Simple random sampling was used to identify the first study participants in any randomly selected module out of the first 9 participants who arrived at the clinic on any given day. Every ninth pregnant woman who reported to the clinic was approached and requested to take part in the study. If the ninth woman was not eligible to participate in the study, the next one was approached until an eligible participant was recruited. Every ninth interval was arrived at by considering that an average of 100 pregnant women are seen on a daily basis and the study duration was 3 months, therefore a total of 6000 women attend the clinic over 3 months. This was then divided by the calculated study sample, N=667, arriving at the ninth interval. Participants were selected from the antenatal register after they had been registered. On an average 12 participants were recruited daily. At any point during the study procedure, the participant had the right to withdraw from the study or to refuse to answer any question. This had been accounted for by a 10% attrition rate in the sample size. A total of 7259 women attended ANC at MNH from December 2017 to February 2018. Eight hundred and seven (807) women were approached to participate in the study, among them, 137 women were not included in the study for the following reasons; 93 women were second contacts, 15 were on antibiotics and 29 women did not give consent. Therefore, of 807 pregnant women, 670 were enrolled in the study. Out of these, vaginal samples from 3 women were inadequate and were therefore excluded. The analysis was thus based on 667 participants.

### Data Collection

The purpose and procedure of the study were explained to the participants and those who gave consent and agreed to participate were enrolled in the study. Each woman was interviewed individually and questionnaires were filled in a confidential location within the ANC. A speculum examination was performed and samples taken in the examination room. This was done after they had been seen by the doctors so as to not interfere with the flow of mothers receiving antenatal care. However, where the patients required a pelvic examination by the doctor, it was done simultaneously as samples were being taken. The antenatal cards of the participants were coded using a marker to avoid re-recruitment and their hospital registration numbers documented until the desired sample size was reached. Data was collected by the Principal Investigator and research assistants using a pretested questionnaire. Three (3) research assistants (Nurse Midwife and two intern Doctors) were trained for a day on recruitment, filling and examination procedures before the commencement of the study. The questionnaire was translated to Kiswahili and pretested before commencement of the study by administering it to 10 pregnant women attending ANC at MNH, we assessed whether the women were able to understand the questions and who they would be comfortable with asking the questions (health care provider/social worker/member of the committee/research staff not working at the facility) and whether they would answer the questions truthfully to that person. We tried to ensure that this group of 10 women was representing the population of women who received care at the clinic by selecting women of different age groups, from different parts of Dar e Salaam (rural/urban), with different levels of education and at various gestational age. The questionnaire was supplemented by important obstetric data relevant for the study from the antenatal card. Collected information was; age, occupation, gravidity, parity, marital status, education levels, information on the presence and type of discharge, history of PROM, history of Pre-Term Delivery (PTD), history of miscarriage, information regarding multiple sexual partners, Life Time Sexual Partners (LTSP), age of sexual debut, vaginal douching, history of STI, HIV infection, smoking and maternal stress. HIV status of women attending ANC at MNH is routinely checked after counselling using the rapid test by a nurse counsellor. Vaginal examination was done and samples were mainly collected and delivered to the laboratory by the Principal Investigator, on days when Principal Investigator was not available, it was done by research assistants. Maternal stress was assessed using the Cohen perceived stress scale (see appendix). This is a widely used 14 item self-report scale which measures the degree to which a respondent appraises her life as being stressful. Questions were asked about feelings and thoughts during the past one month using the Likert scale and questionnaire filled by the principal and research assistants. Each item is rated on a 5-point scale ranging from never (0) to almost always (4). Positively worded items are reverse scored, and the ratings are summated to obtain the stress score.^21^ The levels of stress were grouped as low (<19), low/moderate (20–24), moderate/high (25–29) and high (>29) as per the study done by Culhane et al.^20^

### Collection of Specimen

All participants underwent a standard sterile speculum examination. This was a sterile procedure done mainly by the principal investigator in the examination room with a good light source. The participants were positioned in a lithotomy position, and after wearing sterile gloves, sterile disposable Cusco's specula were used to expose the vaginal walls with no lubricant added. Macroscopic evaluation of the vaginal walls for colour, amount and consistency of the discharge was noted. Thin grey homogenous discharge is characteristic for BV. A vaginal swab was collected using a sterile cotton swab from the posterior fornix of the vagina and then placed in a sterile Stuart's transport medium to maintain moisture and labelled with the patients code number for transport to the laboratory. An evaluation sheet noting the patient's medical record number, patient's code number, and date of examination was filled. The evaluation sheet together with the labelled container were transported to the microbiology laboratory in a cooler box for Gram staining and evaluation each day. The Principal Investigator with the research assistants ensured that the specimens were delivered to the laboratory at the end of the day for processing.

### Reading and Reporting the Smears

In the laboratory, the frosted edge of a glass slide was labelled with the patient's code number and date. The obtained vaginal samples were used to make a thin smear on the labelled glass slide and allowed to air dry. After the smear had dried, it was heat-fixed by passing the slide over a bunsen flame 3 times, cooled off and then Gram stained as follows: The slides were placed on a staining rack, flooded with crystal violet stain and left to stand for 1 min and then gently rinsed under tap water. They were then covered with Gram's iodine solution and left to stand for another 1 min and again washed gently under tap water. After, the slides were tilted slightly and decolourised with acetone until the runoff became clear (1-5 sec), the decolourisation time was adjusted to the thickness of the smear. The excess decolouriser was removed with gentle flow of tap water. The slides were then counter stained by flooding with safranin and allowed to stand for 1 min and then washed off gently under tap water. Finally, the slides were air dried in an upright position. The prepared slides were read and reported. This was done by 2 trained and experienced lab technologists at MUHAS Microbiology Laboratory and in case of a discrepancy, a third opinion was sought and the opinion of the third lab technologist was final. Furthermore, 10% of randomly selected slides were scored by a Microbiologist against a collection of already scored smears. In all, there was a 90% concordance, indicating that scoring was comparable and consistent. Bacterial vaginosis was diagnosed using the Nugent's method. This method is the gold standard and considered optimal because it minimises clinical subjectivity.^15^ Three (3) types of bacteria were evaluated by Gram stain and the results graded using Nugent's criteria. This is a standardised 0–10 point scoring system for evaluation of Gram stained vaginal smears based on 3 morphotypes: large Gram positive rods (*lactobacilli*), small Gram negative/variable rods (G. vaginalis and anaerobic rods) and curved Gram variable rod (Mobiluncus species). A score of 0–3 is considered normal, 4–6 intermediate, and 7–10 positive for BV.^15^ Normal and intermediate are considered as no BV whereas a score of 7 and above is considered to be positive. The results were recorded in the evaluation sheet containing the patient's code number, registration number, and date of examination.

### Quality Assurance

Laboratory diagnosis of BV is mainly achieved by microscopy. Quality assurance ensured good practice in preparing and reading gram stains, competency of the laboratory technologists, and regular preventive maintenance and set up of the microscopes used. MUHAS standard operating procedures were used for gram staining and interpretation of slides. Reagents were made and provided by MUHAS Microbiology Laboratory and checking the appearance of reagents was done daily. Good laboratory practice requires that the report on the Gram smear should mention the presence or absence of yeast cells which was done in the reporting.

### Data Analysis

Data entry was done after developing the template on SPSS version 23. Before data analysis, quick frequency tables were run to check for consistency and missed data. After data cleaning, frequencies, means and proportions of variables were computed and tests of significant difference or association between independent variables and dependent variable was done using Chi square test, results were recorded as odds ratio with 95% CI and a p value of <0.05 was considered significant. Independent variables were social demographic factors such as age, level of education and marriage status, obstetric factors such as gravidity, parity, PTD and miscarriage and behavioural factors such as douching, LTSP, smoking, maternal stress, age of sexual debut, STI and HIV, and the dependent variable or outcome was BV. Univariate analysis was used to calculate frequencies and proportions, bivariate analysis to see the association of selected exposure variables with the outcome variable and multivariable analysis to check the association of possible factors with the BV by adjusting for potential confounders.

### Ethics Approval and Consent to Participate

This study received ethical approval from Muhimbili University of Health and Allied Sciences (MUHAS) Senate Research and Publication committee as well as permission from the Executive Director of MNH. Written informed consent was obtained from the participants prior to their enrolment in the study. Confidentiality was maintained. All participants were informed of their results and participants who were found to be positive for BV, or if found to have vaginal candidiasis were treated as per MNH protocols after communicating with the doctor taking care of the patient.

## RESULTS

Figure 2 shows Gram stain results using the Nugent's criteria. 178 (26.7%) participants were diagnosed to be positive for BV while 489 (73.3%) were found to be negative for BV, (which included both negative and intermediate scores).

**FIGURE 1.**
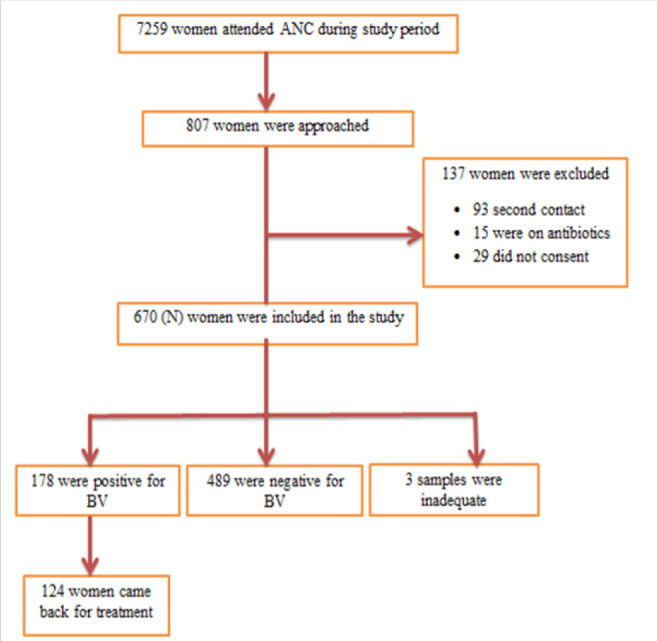
Flow Chart of Patient Recruitment and Follow Up

**FIGURE 2.**
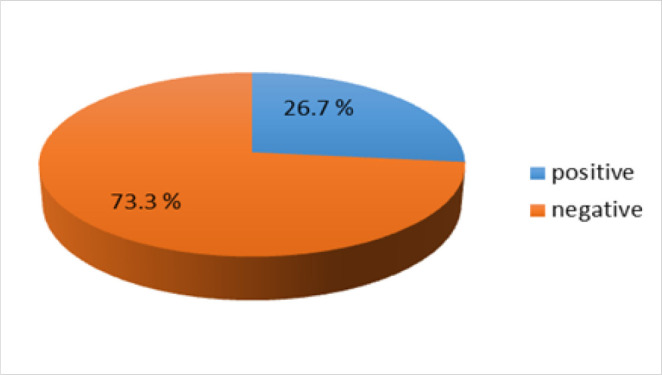
Prevalence of Bacterial Vaginosis among Pregnant Women attending ANC at MNH

Table 1 shows the association of BV with socio-demographic and obstetric factors. Age, education levels, gravidity and parity were significantly associated with BV (*P <0.05*)

**TABLE 1:** Socio-Demographic and Obstetric Factors Associated with Bacterial Vaginosis among Pregnant Women Attending ANC at MNH, (N=667)

Predictors	Total	Bacterial Vaginosis		*P value*
		Positive n (%)	Negative n (%)	
**Age group (years)**				
< 20	56	17(30.4)	39(69.6)	.018
21–29	369	112(30.4)	257(69.6)	
≥30	242	49(20.2)	193(79.8)	
**Marital status**				
Married	481	128(26.6)	353 (73.4)	.944
Unmarried	186	50 (26.9)	136 (73.1)	
Type of marriage^*^				
Polygamous	26	3(11.5)	23(88.5)	.074
Monogamous	455	125(27.5)	330(72.5)	
**Education level**				
Primary and less	249	110(44.2)	139(55.8)	<.001
Secondary and above	418	68(16.3)	350(83.7)	
**Occupation**				
Unemployed	241	69 (28.6)	172 (71.4)	.682
Employed (public or private)	157	41 (26.1)	116 (73.9)	
Self employed	269	68 (25.3)	201 (74.7)	
**Gravidity**				
Primigravida	220	47(21.4)	173(78.6)	.029
Multigravida	447	131(29.3)	316(70.7)	
**Parity**				
Nulliparous	278	58(20.9)	220(79.1)	.004
Parous	389	120(30.8)	269(69.2)	
**History of miscarriage**^#^				
No	292	81 (27.7)	211 (72.3)	.318
Yes	155	50 (32.3)	105 (67.7)	
**History of PTD**^§^				
No	333	98 (29.4)	235(70.6)	.140
Yes	56	22(39.3)	34(60.7)	

PTD – Preterm Delivery,

* N=481, as only 481 participants were married,

# N = 447, is equal to the number of women who were multigravida and

§ N = 389 is equal to the number of women who were parous i.e. women who had at least one delivery above the age of viability (28+weeks).

Table 2 shows that there was a statistically significant association (* P<0.05*), between BV and vaginal douching, HIV status, history of STI, LTSP and age at first coitus In the bivariate analysis (Table 3), age (COR 1.71; 95% CI, 1.16–2.52), level of education (COR 4.08, 95% CI, 2.84–5.84), gravidity (COR, 1.52, 95% CI; 1.04–2.23), parity (COR 1.69, 95% CI; 1.18–2.42), vaginal douching (COR 2.89, 95% CI; 1.96–4.27), HIV status (COR 9.37, 95%CI; 4.12–21.28), history of STI (COR 2.49 95% CI; 1.46–4.25), LTSP (COR 2.76, 95% CI; 1.68–4.54) and age of first coitus (COR 3.19, 95% CI; 2.24–4.56) were significantly associated with BV. After adjusting for confounders in multivariate analysis, factors that were found to be significantly associated with BV were; age of between 21 to 29 years (AOR, 2.22, 95%CI; 1.45–3.49), education level (AOR 3.97, 95% CI; 2.63–5.98), vaginal douching (AOR 3.68, 95% CI; 2.35–5.76), HIV status (AOR 6.44, 95% CI; 2.62–15.88), STI infection (AOR 2.34, 95% CI; 1.25–4.37), more than one LTSP (AOR 2.69, 95% CI; 1.53–4.74) and age of less than 18 years of first coitus (AOR 2.16, 95% CI; 1.42–3.30). Gravidity and parity was not significantly associated with the outcome.

**TABLE 2:** Behavioural and Clinical Factors Associated with Bacterial Vaginosis among Pregnant Women Attending ANC at MNH, (N= 667)

Predictors	Total	Bacterial Vaginosis		P value
		Positive n (%)	Negative n (%)	
**History of smoking before current pregnancy**				
No	646	174(26.9)	472(73.1)	.421
Yes	21	4(19.0)	17(81.0)	
**Vaginal douching**				
No	273	42(15.4)	231(84.6)	<.001
Yes	394	136(34.5)	258(65.5)	
**HIV status**				
Negative	635	154(24.3)	481(75.7)	<.001
Positive	32	24(75.0)	8(25.0)	
**History of STI**				
No	605	150(24.8)	455(75.2)	.001
Yes	62	28(45.2)	34(54.8)	
**Maternal stress**				
Low stress	294	72(24.5)	222(75.5)	.391
Low moderate	274	74(27.0)	200(73.0)	
Moderate high	86	29(33.7)	57(66.3)	
High stress	13	3(23.1)	10(76.3)	
**LTSP**				
1	153	21(13.7)	132(86.3)	<.001
2+	514	157(30.5)	357(69.5)	
**Age at first coitus**				
< 18 years	227	96(42.3)	131(57.7)	<.001
≥ 18 years	440	82(18.6)	358(81.4)	

LTSP – lifetime sexual partners, STI – sexually transmitted infections.

**TABLE 3:** Bivariate & Multivariate Analysis of Factors Associated with Bacterial Vaginosis among Pregnant Women Attending ANC At MNH, (N=667)

Predictors	Bacterial vaginosis n (%)	Bivariate analysis; COR (95% CI)	Multivariate analysis; AOR (95% CI)
**Age group (years)**			
< 20	17 (30.4)	1.717 (0.896, 3.289)	1.544 (0.717, 3.325)
21–29	112 (30.4)	*1.717 (1.169, 2.520)	*2.224(1.415, 3.494)
≥30	49 (20.2)	1	1
**Education level**			
Primary and less	110 (44.2)	*4.087 (2.840, 5.842)	*3.975 (2.639, 5.986)
Secondary and above	86 (16.3)	1	1
**Gravidity**			
Primigravida	47 (21.4)	1	1
Multigravida	131 (29.3)	*1.526 (1.042, 2.234)	0.732 (0.321, 1.671)
**Parity**			
Nulliparous	58 (20.9)	1	1
Parous	120 (30.8)	*1.692 (1.180, 2.427)	1.290(0.820, 2.027)
**Vaginal douching**			
No	42 (15.4)	1	1
Yes	136 (34.5)	*2.899 (1.965, 4.277)	*3.685 (2.356, 5.765)
**HIV status**			
Negative	154 (24.3)	1	1
Positive	24 (75.0)	*9.370 (4.125, 21.285)	*6.442 (2.612, 15.887)
**History of STI**			
No	150 (24.8)	1	
Yes	28 (45.2)	*2.498 (1.466, 4.257)	*2.343 (1.254, 4.377)
**LTSP**			
1	21 (13.7)	1	1
2+	157 (30.5)	*2.764 (1.681, 4.545)	*2.698 (1.536, 4.740)
**Age at first coitus**			
< 18 years	96 (42.3)	*3.199 (2.241, 4.569)	*2.169 (1.423, 3.306)
≥ 18 years	82 (18.6)	1	1

COR - crude odd ratio, AOR - adjusted odd ratio, LTSP - life time sexual partners, STI - sexualy transmitted infections, PTD - preterm delivery, *statistically significant

## DISCUSSION

The prevalence of BV in this study was noted to be high (26.7%) which can be explained by the fact that certain behavioural factors such as douching are practiced by majority of the women in Dar es Salaam.^22^ Higher prevalence has also been reported in several sub Saharan countries including Nigeria, Botswana, Kenya, and Zimbabwe.^6,7,23,24^ In contrast, lower prevalence was reported in Portugal (3.9%), Burkina Faso (6.2%), and India (8.6%).^5,25,26^ This vast difference in prevalence across the globe is presumably due to environmental, behavioural, socioeconomic status and stressor differences in the geographical variation.

The highest prevalence of BV occurred among women in their 20s. Similar findings were reported in studies conducted in Nigeria whereby women aged 21–30 were predominantly diagnosed to have BV as compared to other age groups.^23,27^ In comparison, a French population based study reported maternal age of less than 20 years to be significantly associated with BV as compared to older women.^28^ Others have reported highest prevalence of BV among women aged more than 30 years.^29^ The common finding in all these studies is that the age groups with the highest prevalence of BV are the most sexually active age group with the highest risk of pregnancies and STIs.^29^ More than 50% of the participants in this study were in their 20s hence this could account for the high prevalence noted among this age group. Considering the urban setting of the study, women below 20 years are less likely to get pregnant due to more awareness, accessibility and availability of contraceptives.^30^

It was noted that women who had attended primary level education and less were more likely to get BV as compared to women who had attended secondary level education and above. In a randomised controlled trial done in France, it was seen that women with primary level education were two fold more likely to get BV.^28^ A study done in Nigeria also noted similar findings whereby lack of western education was associated with increased risk of BV.^31^ This could possibly be explained by the fact that women with low education levels may not be knowledgeable about certain harmful practices such as vaginal douching and may also delay in seeking appropriate treatment for conditions such as STI.

Women who douched during pregnancy were significantly more likely to get BV as compared to women who did not douche. This finding is due to the fact that the majority of participants in this study douched during pregnancy. More than 50% of the participants deemed douching as a good hygiene practice, which to a larger extent disturbed the normal flora of the vagina predis-posing them to BV. Douching has been known to cause disturbance of vaginal chemical balance and microbial normal flora hence leading to overgrowth of BV causing microorganisms.^22^ This finding is consistent with results from several other previous studies.^13,20^

The current study noted a high prevalence of BV among HIV infected women. However the prevalence of HIV among the participants was less than 5% in the current study. Therefore, the results may not be representative of all the population. Studies done elsewhere in the world have also highlighted a significant association of BV with HIV infection.^32,33^ This is probably due to immunosuppression caused by HIV infection which predisposes the women to infections such as BV. On the other hand, it has been noted that BV increases susceptibility to HIV infection. Therefore, interventions to reduce the occur-rence of BV may have an impact on the spread of HIV at a population level.

Women with a history of STI were noted to have a twofold increased risk of getting BV as compared to women who had no STI. This finding is consistent with a study done among pregnant Danish women where by women with a history of STI such as Neisseria gonorrhoea and Chlamydia trachomatis had an increased risk of getting BV as compared to women who did not have any STI.^34^ This was also noted in another study conducted in England, where women with bacterial STI had a higher risk of getting BV.^35^ The association between STI and BV could presumably be due to high-risk sexual behaviours such as multiple sexual partners among these women. Though, having previously experienced the symptoms of STI may make them more aware of vaginal abnormalities and thus seek treatment earlier.

The current study found that having more than one LTSP significantly increased the risk of getting BV. This finding is consistent with other previous studies.^9,36^ In the current study, a significant relationship between more than one LTSP and BV was established as more than 3 quarters of the participants had more than one LTSP. In comparison, most of the studies conducted to establish the association of LTSP and BV, the study population was of women who are not pregnant. This is one of the few studies to do so on women during pregnancy. It has been suggested that increased number of LTSP predisposes to BV by causing the vaginal flora to become unstable.^37^ Changes in the vaginal environment induced by sexual intercourse with a new partner may increase susceptibility to abnormal colonisation in certain women due to disruption of the woman's already established vaginal flora. Coitus alters the physiochemical vaginal environment thereby affecting the vaginal microflora. In particular, it has been shown that the alkaline prostatic content of the ejaculate raises the vaginal pH and this favours the growth of the anaerobes.^38^

Early age of sexual debut before 18 years was found to be significantly associated with BV. This finding is consistent with a study conducted in Zimbabwe, where by sexual debut before the age of 20 years was found to be the strongest predictor of vaginal infections.^7^ The reason for this is not quite clear. However, it could be presumed that women who have an early sexual debut are likely to be more sexually active or have more sexual partners. In addition, immaturity of the genital tract making it to be more susceptible to vaginal infection.

The major strength of the current study was that it was conducted in the largest tertiary care hospital in Dar es Salaam and therefore included participants from most parts of Dar es Salaam.

This strength is also based on the large sample size. The limitation of this study was that self-reported risk behaviours and history of STI might have been under reported due to social acceptability. Despite this limitation, the data is reliable and can be used as proxy to predictors of BV among pregnant women.

## CONCLUSION

Age below 30 years, primary education level and below, vaginal douching, HIV infection, STI, more than one lifetime sexual partners and early age of sexual debut were found to be significant predictors of BV. The high prevalence of BV in our population should necessitate policy makers to include screening and treatment of BV in ante-natal care package in the future, as BV is associated with significant maternal and neonatal morbidity and mortality. Women should also be educated on harmful effects of certain behavioural practices such as vaginal douching which lead to alteration of the vaginal flora thus predis-posing to BV. In addition, symptoms of BV such as abnormal vaginal discharge during pregnancy are inconsistent, under reported and often overlooked. Therefore, a high-risk approach can be used for screening and treatment of asymptomatic women.
